# Perceptions, attitudes and knowledge of evidence-based medicine in primary care in Spain: a study protocol

**DOI:** 10.1186/1472-6963-9-80

**Published:** 2009-05-15

**Authors:** Pablo Alonso-Coello, Ivan Solà, Rafael Rotaeche, Ana Isabel González, Mercè Marzo-Castillejo, Arturo Louro-González, Ricard Carrillo, Paola Velázquez, Guillermo García-Velasco, Carlos Calderón

**Affiliations:** 1 Iberoamerican Cochrane Center, Hospital Sant Pau, Sant Antoni Maria Claret 171, Barcelona, Spain; 2 CIBER de Epidemiología y Salud Pública (CIBERESP), Spain; 3 Centro de Salud de Alza, Donostia-San Sebastián, Spain; 4 Centro de Salud Vicente Muzas, Área 4, Madrid, Spain; 5 Àmbit d'Atenció Primària Costa de Ponent, Barcelona, Spain; 6 Servicio de Atención Primaria de Cambre, Cambre, Coruña, Spain; 7 EAP La Florida Sud, L'Hospitalet de Llobregat, Barcelona, Spain; 8 Consultas Médicas y Psicológicas, Barcelona, Spain; 9 Centro de Salud La Calzada II, Gijón, Asturias, Spain

## Abstract

**Background:**

The philosophy of evidence-based medicine (EBM) was introduced in the early 90s as a new approach to the practice of medicine, using the best available evidence to make decisions about health care. Despite ongoing controversy, EBM has developed enormously and physicians' attitude towards it is generally positive. Nevertheless, in Spain little is known about this topic. We will therefore undertake a study to explore perceptions, attitudes and knowledge about EBM among primary care physicians.

**Methods and design:**

A mixed-method study combining qualitative and quantitative designs will target family practitioners in Spain with the objective of evaluating current attitudes and perceptions about evidence-based medicine. The project will consist of two phases: a first phase running focus groups to identify perceptions and attitudes of participants, and a second phase assessing their attitudes and knowledge about EBM by means of a survey. Both phases will explore these issues in three different subgroups: family practitioners, with or without previous formal education in EBM; members of working groups that formulate healthcare recommendations; and physicians in charge of training family practice residents. Additionally, we will undertake a systematic review to identify and synthesize the available evidence on this topic.

**Discussion:**

The study will identify and gain insight into the perceived problems and barriers to the practice of evidence-based medicine among general practitioners in Spain. The project will also evaluate the main knowledge gaps and training needs, and explore how evidence-based medicine is being taught to family medicine residents, the medical practitioners of the future. Our results will aid researchers and health care planners in developing strategies to improve the practice of evidence-based medicine in our country.

## Background

Evidence-based medicine (EBM) was introduced in the early 90s as a new paradigm in the teaching and practice of medicine, deemphasizing unsystematic clinical observations and pathophysiological rationale [1]. Its main proponents recognized the value of traditional medicine but emphasized the need for new skills: formulation of well-structured questions, search for the best available evidence, and critical interpretation of the design and execution of studies, as well as results. EBM has since developed tremendously, promoting fundamental advances in accessing and understanding information, improving the development of pre-appraised information products and fostering the role of patients' values and preferences in clinical practice [2].

Despite continuing discussion and debate [3-5], EBM is generally accepted, though not necessarily incorporated, as an integral component of clinical practice by medical doctors, nurses, pharmacologists, health management teams, and a long list of allied health professionals. Recent research has shown that physicians' general perception and attitude to EBM is positive [6-13]. Despite this, there is still a need to improve research skills and critical appraisal [6,7] and a certain degree of rejection towards EBM's reductionist focus is evident [8-13]. Furthermore, implementation of new information into daily clinical practice is slow.

In Spain virtually no information is available on physicians' perceptions and attitudes towards EBM. In primary care this topic is particularly complex, little studied and misunderstood, and its true impact in this setting is uncertain. The new Training Program in Family Medicine emphasizes that learning EBM and using Internet resources are paramount for clinical practice [14]. Nevertheless, studies in other countries and areas indicate that residents encounter major difficulties in accessing and implementing EBM in their training and that there is a lack of tutors proficient in this area [15-17]. Furthermore, it is not known how tutors of Family Medicine in training units in Spain perceive EBM.

In semFYC, Spain's main family practice society (Spanish Society of Family and Community Medicine), more than 30 working groups prepare and widely distribute recommendations for primary care professionals. EBM knowledge and skills among these working group members has never been explored. Taking all this into account, a study examining perceptions, attitudes and knowledge of EBM among general practitioners, working group members, and tutors of family practice, is relevant and necessary to bring some light to these issues, and to determine the main barriers preventing the use of EBM in Spain.

## Methods and design

The study will include two clearly differentiated phases and methodologies. During Phase I, with a qualitative design, we will run focus groups to gain insight into the perceptions and attitudes of family practitioners, working group members and tutors towards EBM. In a second quantitative stage, Phase II, we will evaluate knowledge and general attitudes towards EBM of these populations, by means of a questionnaire. Family practitioners and working group members will be gathered from the database of the main family practice society in Spain (semFYC) that includes 20,000 physicians. The study was approved by the Hospital Sant Pau Ethics Committee.

### Phase I: Qualitative Study

Qualitative research methods have proved to be extremely useful in the health care field, providing in-depth knowledge concerning perceptions, beliefs and values of the persons and groups involved [18,19]. These methods provide researchers with the opportunity to explore potential hypotheses as to why participants hold the views they do. We thus considered this approach would be appropriate for the study objectives.

Taking into account how important it is to evaluate behavior and attitudes of our target populations (family practitioners, tutors and working group members), we consider the group interview will be the most adequate technique to gather for information. Known as focus groups [20,21] or discussion groups [22,23], qualitative research techniques explore dynamic interactions among group members, consider issues that may underlie individual preferences and explore areas of consensus and disensus. In this study the development and use of a focus group will reveal participants' experiences and views concerning EBM

#### 1. Planning and structuring focus groups

A group of experts will determine the basic topics the focus groups are to explore. They will be based on some of the most relevant and controversial issues related to the development and implementation of EBM in clinical practice. Once these areas have been agreed upon a final semi-structured questionnaire will be decided and prepared for the focus groups. The content of this questionnaire might be modified depending on the findings that arise during the development of the focus groups [24].

A minimum of eight groups will be necessary (four groups of family practitioners, two of working group members and two of family medicine teaching unit staff) to reach the point of saturation needed for obtaining and analyzing the information. Each group will consist of between 9–11 people and will be selected taking into account their structural inter-group representation: origin of the populations, degree of knowledge and experience in EBM, place of work, age and sex [23]. At the same time, we will try to guarantee the intra-group homogeneity needed to promote discussion and interaction between participants.

#### 2. Recruitment and participants

The researchers will gather a purposefully selected sample, choosing participants according to their representativeness and capacity to provide rich and varied information in relation to the study objectives. With the study objective in mind, while recruiting we will explain clearly to the potential participants that knowledge and quality of clinical practice will not be evaluated. Confidentiality assurance will be given concerning all group contents and the independent nature of the study will be emphasized. We will recruit potential participants by means of a formal letter of invitation and travel costs will be reimbursed.

The focus groups will be drawn from four autonomous communities in Spain: Galicia, the Basque Country, Madrid and Catalonia. There will be eight focus groups in total, consisting of four groups of family practitioners, two groups of working group members and two groups of family practice tutors. The family practitioner groups will be further divided into two: those who have some kind of formal training in EBM and those who do not. By formal training we will consider having attended any course, workshop or activity related to EBM in the last five years. The inclusion criterion will be present engagement in clinical work for family practitioners, being an active member of a working group in the case of working group members, or being in charge of residents at the present time in the case of family practice tutors.

#### 3. Focus group moderating

An interviewer and a collaborator will be needed to moderate each focus group. In as far as possible, the interviewer will not be recognized as an EBM expert and care will be taken to avoid commenting and giving opinions about the topic. The interviewer will introduce the study's objective, explain the need to record the session and reassure participants about the confidentiality of all contents. The interviewer will additionally ask participants for their informed consent before the study starts.

After collecting some basic demographic information, the interviewer will moderate the group by raising questions and will lead the group throughout the session so as to maintain focus and cover the programmed issues. The collaborator will observe the group dynamics and record the results of group work, including non-verbal language. Group sessions will be designed to be flexible and open to possible new topics and issues raised by participants. The two investigators will meet together after finishing the groups to detail the experiences in a diary. This information will be analyzed in conjunction with the data obtained by taping the groups [24].

#### 4. Data organization and validation

All information generated will be audio-taped and stored digitally. Audio-tapes will be transcribed literally and the information collected by the interviewers and observers will be incorporated. To guarantee accuracy, transcriptions will be verified by the interviewers of each group.

The information obtained will be analyzed following the sociological analysis discourse model [25][26]. Themes and patterns will be identified and coded, keeping in mind the texts as a whole and the contexts in which they have been produced. Each step in the configuration of the potential explicative axes requires new purposive and iterative readings of texts to legitimate and corroborate the interpretation of findings [27]. As distinguished from other analysis models, in this case the reading and organization of data [28] does not rely on the fragmentation of texts but on the interpretation of the different discursive positions and the meaning of participants' interventions. We will use the MAXqda software to organize and contrast the main explanatory axes with the empirical reference of texts, and to select the most representative verbatims. The process and provisional analysis will be triangulated and agreed by consensus among investigators and the results will be checked with participants in order to improve the validity of the study [29]. The reading and organization of the data [27] does not rely on the fragmentation of texts but on the different discursive positions of participants and the main explanatory axes of their interventions.

#### 5. Systematic review of perceptions and attitudes

In parallel with the running of the focus groups we will undertake a formal systematic review [30,31] to explore and evaluate the available evidence on this topic. Our inclusion criteria will be any type of design (qualitative or quantitative) evaluating attitudes and perceptions of family physicians about EBM. We expect to find mainly focus groups, in depth interviews and general surveys that will be explored separately and as a whole. We will evaluate the quality of the design and execution of the studies and look for areas of consensus and disensus to articulate our main findings and discussion.

### Phase II: SURVEY

#### 1. Design of the questionnaire

We will use a single questionnaire divided in four main blocks and adapted from that developed by McColl et al in the United Kingdom [13]. The first part will consist of 10 sections that evaluate professionals' attitudes towards EBM, by means of a Likert scale. We will contrast the original structure and items with the information obtained in the focus groups (phase I) in order to refine and complete its content and make it more representative of our local circumstances. The rest of the survey will explore preferences concerning strategies used to move EBM from theory to practice, physicians' knowledge of EBM, accessibility to EBM in their local practices and use of tools needed to put EBM methodology into practice.

#### 2. Sample

The questionnaire will be sent via e-mail to all potential participants in the three main subgroups of interest (family physicians, working group members and tutors) with a letter inviting them to collaborate and a link to an electronic version of the questionnaire. A reminder will be sent to those who have not responded within two weeks. If there is still no response, they will be contacted by fax or phone at their place of work. For those professionals without an e-mail address, the questionnaire will be sent by ordinary mail to their work addresses, with a stamped return envelope to send back the completed questionnaire. A reminder will be sent after four weeks if no reply is received. As for the group without a known e-mail address, we will contact those who have not responded by fax and phone to confirm receipt of the questionnaire and interest in participating. An investigator's contact details will be provided for participants to raise questions or doubts about the questionnaire or the project.

We estimated a sample size of 600 respondents needed to answer our main objective (percentage of favourable responses towards EBM) of the survey in the family physicians group, with a precision of 4%. To obtain this precision we dichotomised the first item of the survey (being favourable or not to EBM) assuming maximum variability (50% of responses favourable to EBM). A confidence interval of 95% will be applied to the percentage of favorable responses. In the case of working group members and tutors we will be sending the survey to everyone included in the society database.

#### 3. Recruitment

The practicing family doctor sample will be selected randomly and grouped by age, sex and region. One thousand questionnaires will be sent out to family doctors. A minimum response rate of 60% will permit 600 valid responses for the analysis. A higher response rate for the other two groups evaluated, working group members and tutors, is expected. Despite a smaller sample size and despite a higher response rate, it is expected that the final degree of accuracy will be similar to that of the family practitioners group, given their expected greater favorable response rate to EBM.

#### 4. Analysis

The 95% confidence level will be applied to the main outcomes. The between-group comparison will be described for qualitative outcomes by means of contingency tables and inferences with the Chi Square Test or Fisher's Exact Test. Quantitative outcomes will be described using means and their corresponding standard deviations. Inferences will be made using an analysis of variance (ANOVA). If we encounter clearly defined application problems (abnormality and heteroscedasticity) the corresponding non-parametric test will be used. All analyses will be conducted using a bilateral approach. We will use the usual 5% significance level (α = 0.05) and the SPSS statistical package, version 11.5, to run the proposed analysis.

## Discussion

This study will: a) shed light on the perceived difficulties and barriers to the comprehension and application of EBM; b) illustrate the needs for discussion, education and use of EBM to improve clinical practice of family doctors and; c) inform how EBM philosophy can be transmitted to future generations of family doctors as an approach to their education and clinical practice.

We will undertake our study in a wide and diverse population of family practitioners. This will make our results more relevant and generalisable. The results will give us a broad picture of how EBM is performing in our country. Additionally, we will be able to compare findings with the research published to date. We will also be able to explore the level of knowledge and use of EBM in clinical practice, and develop recommendations and clinical teaching strategies.

In brief, our study will include a systematic review about the topic that will give an overall picture of the situation internationally, a qualitative phase to explore attitude and perceptions, and a final national survey to evaluate these inform how EBM use. This project will be one of the the first in Spain to address this important question and will inform family practitioners and decision makers about the needs and challenges in this exciting field.

## Abbreviations

ANOVA: analysis of variance; EBM: evidence based medicine; semFYC: Spanish Society of Family and Community Medicine; SPSS: Statistical Package for the Social Sciences.

## Competing interests

The authors declare that they have no competing interests.

## Authors' contributions

IS, CC, RR, and PA-C participated in the conception and design of the protocol and drafted a first version. All authors participated revising it critically for important intellectual content and have given final approval of the version to be published.

## Pre-publication history

The pre-publication history for this paper can be accessed here:


